# In the Direction of an Artificial Intelligence-Enabled Monitoring Platform for Concrete Structures

**DOI:** 10.3390/s24020572

**Published:** 2024-01-16

**Authors:** Gloria Cosoli, Maria Teresa Calcagni, Giovanni Salerno, Adriano Mancini, Gagan Narang, Alessandro Galdelli, Alessandra Mobili, Francesca Tittarelli, Gian Marco Revel

**Affiliations:** 1 Department of Industrial Engineering and Mathematical Sciences, Università Politecnica delle Marche, 60131 Ancona, Italy; m.t.calcagni@staff.univpm.it (M.T.C.); g.salerno@pm.univpm.it (G.S.); gm.revel@staff.univpm.it (G.M.R.); 2 Department of Information Engineering, Università Politecnica delle Marche, 60131 Ancona, Italy; a.mancini@staff.univpm.it (A.M.); g.narang@pm.univpm.it (G.N.); a.galdelli@staff.univpm.it (A.G.); 3 Department of Science and Engineering of Matter, Environment and Urban Planning, Università Politecnica delle Marche, 60131 Ancona, Italy; f.tittarelli@staff.univpm.it; 4 Institute of Atmospheric Sciences and Climate, National Research Council (ISAC-CNR), 40129 Bologna, Italy

**Keywords:** self-sensing concrete, monitoring, electrical impedance, monitoring platform, Artificial Intelligence, vision systems, crack detection, early warning

## Abstract

In a seismic context, it is fundamental to deploy distributed sensor networks for Structural Health Monitoring (SHM). Indeed, regularly gathering data from a structure/infrastructure gives insight on the structural health status, and Artificial Intelligence (AI) technologies can help in exploiting this information to generate early warnings useful for decision-making purposes. With a perspective of developing a remote monitoring platform for the built environment in a seismic context, the authors tested self-sensing concrete beams in loading tests, focusing on the measured electrical impedance. The formed cracks were objectively assessed through a vision-based system. Also, a comparative analysis of AI-based and statistical prediction methods, including Prophet, ARIMA, and SARIMAX, was conducted for predicting electrical impedance. Results show that the real part of electrical impedance is highly correlated with the applied load (Pearson’s correlation coefficient > 0.9); hence, the piezoresistive ability of the manufactured specimens has been confirmed. Concerning prediction methods, the superiority of the Prophet model over statistical techniques was demonstrated (Mean Absolute Percentage Error, MAPE < 1.00%). Thus, the exploitation of electrical impedance sensors, vision-based systems, and AI technologies can be significant to enhance SHM and maintenance needs prediction in the built environment.

## 1. Introduction

Structural Health Monitoring (SHM) tools are fundamental to optimise the life cycle of a structure or an infrastructure, enhancing both the quality of its performance and the intervention costs needed for its management [1]. Many different technologies can be applied in this context, both hardware and software ones. For example, it is possible to mention accelerometers [2], strain sensors [3], vision systems [4], and electrical impedance sensors [5]. Sensing technologies and data processing techniques rapidly evolve, giving more and more opportunities for the smart monitoring of structures, which is sometimes different from traditional sensors. Sabato et al. [6] highlighted the potentialities of non-contact systems (e.g., thermal cameras, laser-based systems, etc.) for large structures (requiring dedicated extra-large strain sensors—e.g., Sun et al. [7] proposed wide-range Fiber Bragg Grating sensors, which proved to be linear and stable), ensuring high spatial resolution as well as effectiveness in cost and ease of use. Also, vibration-based methods are flexible enough to be scaled to different application fields [8]. Sadhu et al. reviewed SHM techniques based on Building Information Modelling (BIM) and virtual reality [9], underlining the relevance of the acquired big data and the necessity of appropriate data management systems to deal with them. However, electrical impedance sensors play a pivotal role since they are frequently combined with self-sensing materials [10]: conductive additions are inserted in the mix design to enhance the electrical conductivity of the material. In this way, the passage of electric current is eased and the utilization of sensors measuring electrical impedance or conductivity is supported. Different types of electrical sensors can be applied, but the use of a 4-wire configuration and of alternating current (AC) is fundamental to avoid the polarization of the electrode–material interface and of the material itself, respectively [11,12]. Moreover, different types of sensors exist, such as embedded or surface electrodes; however, for continuous monitoring, the latter surely provides a more robust and accurate system with respect to the former [13] (whose contact with the material is difficult to maintain for long times). In a seismic context the application of self-sensing materials and related electrical impedance sensors can be particularly beneficial since it enables the structure to perceive external loads, hence supporting early warning generation and easing prompt interventions.

Owing to the significant progress in SHM tools, integrating different sensors providing an insight on the health of buildings has become commonplace in contemporary construction. Monitoring systems undoubtedly outperform inspection strategies [5] since they provide regular data in a continuous manner and usually are automated. This avoids the presence of operators, which could be unpractical and also dangerous in certain scenarios (e.g., after an earthquake) [14,15]. Moreover, distributed sensor networks can provide data on multiple points of a structure, depicting the global scene in a more accurate and complete manner while also distinguishing among different parts of the same building, for example.

In a seismic context, monitoring the structural health status of a structure/infrastructure, especially a critical one, is pivotal [16]. Early warnings alerting stakeholders of eventual abnormalities can support timely and efficient interventions as well as the design of a priority map of actions to be undertaken during emergencies [17]. When an issue is evidenced by data from monitoring, further inspections can be put in place. The evaluation of cracks plays an important role; traditional measurement methods include steel ruler, magnified graticule, plastic tell-tale, glass tell-tale, brass screw, calliper, and displacement transducers [18,19,20]. Alternative methods have been recently investigated, like capacitive dense sensor array and vision system-based methods for detection, quantification, and localization of the defect [21,22,23]. They are advantageous since they can be used as semi-automatic systems for a quick evaluation of the fissure defects in construction products, hence increasing efficiency in inspection operations.

In this context, tools supported by Artificial Intelligence (AI) and Internet of Things (IoT) technologies enable action in near real-time, while also providing data remotely and alerting the interested parties to support decision making with accurate objective data [24]. To this aim, evaluating the measurement uncertainty of the employed sensors is fundamental. Indeed, AI algorithms have also recently been widely employed both for classification and regression purposes in the construction sector, leading to the ability to process vast amounts of sensor data swiftly and accurately, providing real-time insights into a building performance based on regression, and then anticipating any anomalies using classification techniques. Many studies demonstrate the potential of machine learning (ML) and deep learning (DL) models to predict the fragility and vulnerability of structures. They can learn complex patterns and relationships from input data, making increasingly accurate and reliable forecasts. Galdelli et al. [25] addressed the critical issue of predictive maintenance on infrastructures, particularly focusing on high-risk structures such as bridges, dams, and tunnels, by introducing a novel concept that employs a remote machine vision and sensor-based inspection system designed for predictive maintenance of infrastructures. Such approaches involve integrating advanced robotic technologies with computational systems remotely to make decisions on infrastructure health. Numerous sensors in these systems generate other data that can be useful in the additional attributes of the buildings. Statistical methods such as ARIMA (Autoregressive Integrated Moving Average) have been proven effective in monitoring built environments since the development stage and successfully demonstrate capabilities as early warning systems [26]. Further, a comparative study for the specific application of resilience testing of steel structure bridge data indicated that SARIMAX (Seasonal Autoregressive Integrated Moving Average with exogenous factors) is a perfect model for evaluating time series data and performing anomaly detection simultaneously [27]. Even if these statistical approaches are accurate, they are often complex in implementation and offer little flexibility, especially when it comes to incorporating exogenous variables. Indeed, a compromise between model complexity, performance, and features should always be sought. Recently, newer approaches have emerged across various forecasting applications, such as ML and DL [27], overcoming complex implementation limitations while maintaining comparable performance. In the context of ML approaches, Dipietrangelo et al. [28] focused on the application of ML to SHM with a specific emphasis on detecting impacts on an aluminium plate; they used polynomial regression and a shallow neural network. The study involved comparing the models for impact detection in SHM; it was observed that the shallow neural network performed better between the applied ML models; however, optimization is still ongoing. Notably, Prophet is an open-source forecasting approach developed by Facebook and has showcased promising results in many time series prediction studies [29].

A great potential can be seen also in the context of exploiting data from SHM techniques to assess any structural faults in the built environment. There is a need for further exploration of the monitoring capabilities of ML and statistical models in scenarios involving cracking phenomena and the associated forces to comprehensively assess these models’ efficacy in damage prediction. 

The aim of this paper is to present a measurement procedure involving self-sensing materials, hardware sensors, and AI-based software to be integrated in a monitoring platform specifically developed for SHM in a seismic context. This contributes to the field of structural engineering and seismic safety by exploiting self-sensing materials, effective and accurate sensors, and advanced processing techniques to exploit monitoring data for early warning purposes, making structures more resilient towards natural hazards like earthquakes. In particular, loading tests were designed and performed to evaluate the piezoresistive behaviour of scaled concrete beams with self-sensing capabilities; both force and electrical impedance signals were acquired, and the caused damages were objectively quantified through an approach based on a vision system to recognise defects on concrete surfaces according to Steger’s theory [30]. Moreover, the authors evaluated the performance of different AI models in damage prediction (useful for early warning purposes), looking for a compromise between complexity and performance. Thus, the evaluation encompassed considerations on model complexity and effectiveness to determine the most suitable approach for the specific application in enhancing damage prediction accuracy.

This research activity was performed within the national project reCITY (“Resilient City—Everyday Revolution”–PON R&I 2014–2020 e FSC “Avviso per la presentazione di Ricerca Industriale e Sviluppo Sperimentale nelle 12 aree di Specializzazione individuate dal PNR 2015–2020”, identification code ARS01_00592) [31]. The objective of the project is to realize a holistic system, considering social, economic, and technological aspects, aimed at enhancing the resilience of a community in emergency situations. In particular, the authors are involved in the development of a monitoring system based on innovative materials and related sensors to enhance the resilience of critical structures and infrastructures in a seismic context.

The remainder of this paper is organised as follows: materials and methods of the study are described in Section 2; results are outlined and discussed in Section 3. Finally, the authors provide their final remarks in Section 4.

## 2. Materials and Methods

A total of 6 scaled concrete beams were manufactured with a limestone Portland cement CEM II/A-LL 42.5R as binder at a water/cement ratio of 0.50; half of them (labelled as D, E, and F) were dedicated to the measurement of the fracture load, the others (labelled as A, B, and C) were kept for this study. Coarse gravel (10–15 mm), intermediate gravel (5–10 mm), and calcareous sand (0–8 mm) were used as aggregates. Two conductive additions were employed to lend concrete the self-sensing capability, namely 6 mm long recycled carbon fibres (RCF) supplied by Procotex Belgium SA and biochar filler (BCH) provided by RES Italia. The former were used at 0.05 vol% and the latter at 0.5 vol% on the total mix. The mix design is reported in Table 1. The casting procedure was performed with a concrete mixer; first, the sand and gravels (both intermediate and coarse types) were stirred then cement was added and mixed together. Hence, first BCH and then RCF were included and dispersed. Finally, water was added, and further mixing was carried out. The fresh mixture was poured into prismatic moulds (10 cm × 10 cm × 50 cm).

The two conductive additions used in this work were chosen and dosed based on the results of a previous study (performed within the European H2020 EnDurCrete project, GA n. 760639 [32]). This study led to the patent n. 102020000022024 “Eco-compatible and self-sensing mortar and concrete compositions for manufacturing reinforced and non-reinforced constructive elements, related construction element and methods for the realization of self-monitorable building structures” [33]. Indeed, this combination of conductive additions was also tested within the reCITY project and turned out to be the optimal one to enhance the self-sensing properties of cement-based specimens. The proposed solution can be considered both cost-effective (given their low dosages and since RCF are recycled materials and biochar is a by-product) and efficient in terms of self-sensing ability; moreover, they are in line with the objectives of sustainability and circular economy.

**Figure 1:**
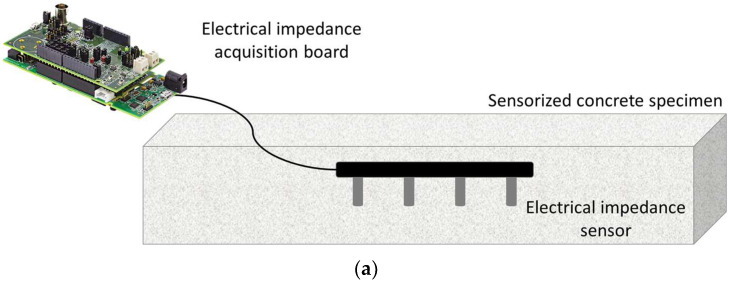

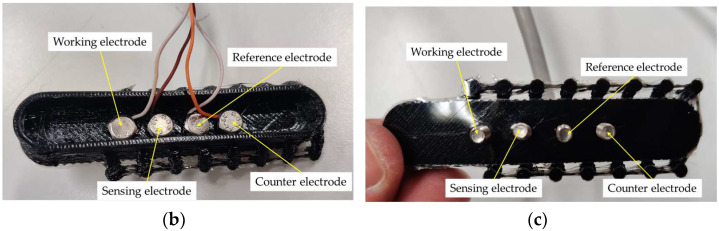
Concrete specimen sensorized for electrical impedance measurement and related acquisition board: (**a**) scheme and pictures of (**b**) bottom and (**c**) top views.

The concrete specimens were cured in environmental conditions (i.e., a temperature of 20 ± 1 °C and relative humidity of 50 ± 5%); during this period, the electrical impedance of the material was measured at 1, 8, 14, 21, and 28 days. The measurements were performed through the sensors embedded in the casting procedure (Figure 1). Moreover, the flexural strength (R_f_) was assessed after 28 days of curing to determine their fracture load and set up the loading tests.

### 2.1. Loading Tests

**Figure 2:**
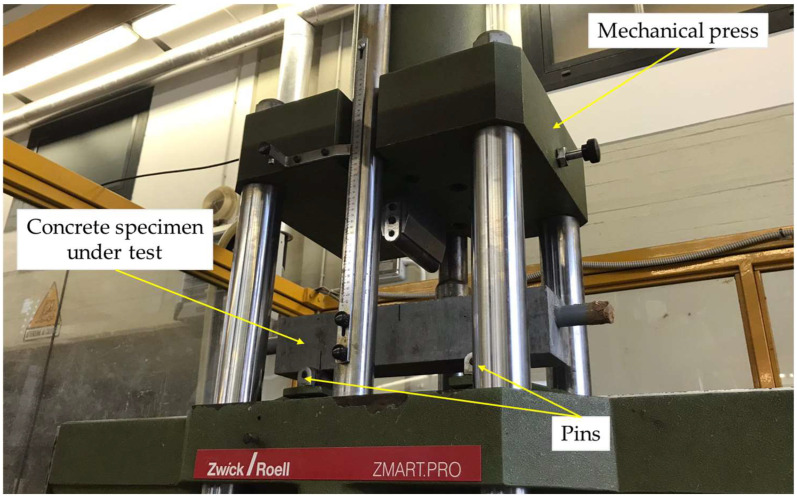
Experimental test setup of loading tests.

After the curing period, the specimens were subjected to flexural loading tests, using a mechanical press (Zwick Roell, Ulm, Germany) with a maximum applicable load of 600 kN. In particular, the scaled beam was positioned on two pins (inter-distance: 30 cm) and the load was applied on the specimen midline. The loading velocity was equal to 0.1 mm/min. Three different load levels were considered: namely, 90% of fracture load (t1), fracture load (t2), and load causing a crack aperture of approximately 1 mm (t3). The applied force was measured with a load cell embedded in the mechanical press; during the load application, electrical impedance measurements were also carried out. The experimental test setup is reported in Figure 2.

### 2.2. Electrical Impedance Measurements

As in the measurements during the curing period, electrical impedance was assessed according to the 4-wire Wenner’s method in alternating current in order to avoid the polarization both at the electrode–material interface and of the material itself, respectively; a single frequency equal to 10 kHz was used for the acquisitions during both curing and loading tests. The external electrodes (i.e., working and counter electrodes) are used to inject the excitation current, whereas the corresponding voltage is measured between the two internal electrodes (i.e., sensing and reference electrodes). The ratio between voltage and current provides the electrical impedance, from which the electrical resistivity can be derived (taking into account the cell constant correction factor, which, however, is quite impractical to be used for in-field applications). It is worth noting that this parameter refers to a limited volume of the material, which corresponds to a hemisphere whose radius is equal to the electrode spacing. The measurements of electrical impedance were performed with a low-cost system based on the AD5940 chip (Analog Devices, Wilmington, MA, USA) embedded in the EVAL-AD5940BIOZ board, which is particularly suitable for distributed sensor networks. The test settings were defined and validated in a previous research activity performed by some of the authors [5]. 

The acquired data were stored in a local database and made available in the FIWARE platform [34] thanks to IoT capabilities of the system. FIWARE is a robust and open-source platform designed to facilitate the development of scalable and interoperable smart applications, making it particularly apt for handling diverse data streams associated with different forecasting models. It provides a standardized set of APIs and components for building smart applications by offering real-time data processing pipelines and a range of reusable enablers. While the collected data were initially processed offline during the model development phase, leveraging FIWARE allowed the realization of real-time IoT data collection for subsequent iterations. This shift to real-time data processing was crucial for accurately optimizing and benchmarking the forecasting models regarding efficiency and reliability. Then, the approach can be easily extended [35] thanks to its dynamicity and responsivity towards data inputs, enhancing the adaptability of the models to changing conditions as if they were collected in real-time. The current pilot study focuses on developing forecasting models and implementing a real-time forecasting pipeline; the development of a warning system is outside the scope of the ongoing investigation. This delineation allows for a focused exploration of the model effectiveness before expanding the scope to incorporate real-time forecasting functionalities in future stages of the project.

The electrical impedance signals were evaluated in relation to the external load applied to the specimen; to this aim, the force data measured by the load cell were considered. The measurement correlation and sensitivity with respect to the applied force was evaluated in the different test times. It is worth noting that the part of the signal corresponding to the crack formation was excluded from these analyses since there is a high peak forming due to the interruption of the electrical current path, and this clearly differentiates from the applied force signal.

### 2.3. Crack Assessment

**Figure 3:**
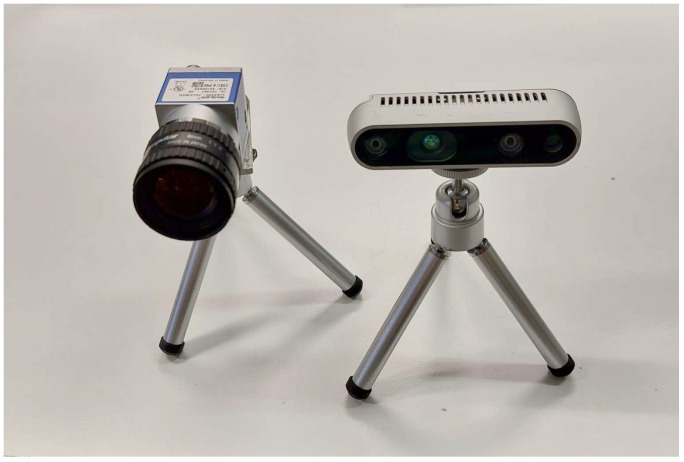
Cracking monitoring toolkit: Basler ace uMED camera (**left**) and Intel RealSense D435i depth sensor (**right**).

In order to have an objective assessment and metrological characterization of the cracks caused by the load tests, concrete fissures were analysed with a vision system consisting of a monochromatic high resolution industrial camera for 2D visible image acquisition (Basler ace uMED, Basler Inc., Ahrensburg, Germany) and a depth sensor (Intel RealSense D435i, Intel, Santa Clara, CA, USA) for depth signal acquisition (i.e., coordinates between the surface of interest and the employed sensor) (Figure 3). In the field of measurements based on vision systems, several limitations must be considered to ensure accuracy and reliability. The resolution of the camera plays a crucial role: a higher resolution offers more details but requires more power and processing time. Conversely, a lower resolution may fail to capture the finest details. Ambient lighting conditions significantly affect the quality of the captured image; inconsistent or inadequate lighting can cause poor contrast and shading, leading to inaccuracies. These factors define the boundaries within which vision systems operate and emphasise the need for careful calibration and environmental control. To prevent these issues, the Basler camera has been chosen for its high resolution and ability to capture details, especially for photos taken closely.

The measurement of the crack aperture was performed using an AI model trained on a real concrete dataset, able to segment the material failure, hence localizing the defect in combination with an algorithm based on Steger’s theory [36] for the detection and measurement of curvilinear structures. To better identify the defects, it is necessary to train the neural network with a consistent dataset of real-life images; hence, a dataset was created with real images of cracks in concrete gathered both from websites and in-field. The neural network used for segmentation and defect identification is UNet [36]. It is worthy to underline that the proposed approach allows the identification of a crack on the picture of the cement-based element, to precisely locate it with sub-pixel resolution, and to assess its aperture width [36]. The visible images were employed for the detection and quantification of the crack aperture, utilizing the image reference system expressed in pixels. Subsequently, the acquired depth values, when integrated with the camera parameters, enabled the conversion to the real-world reference system from pixels to millimetres.

It is worthy to underline that the experimental measurement of the concrete fissure opening was performed after the loading tests and, in particular, after the external load was removed from the specimen surface. This means that a partial closure of the crack happened between the loading test and the crack characterization procedure with the vision systems. The proposed approach can be put in place when the results from the continuous monitoring system evidence some potential issues in the structure and maybe some interventions are needed in the near future.

### 2.4. The Monitoring System and the AI Algorithms

**Figure 4:**
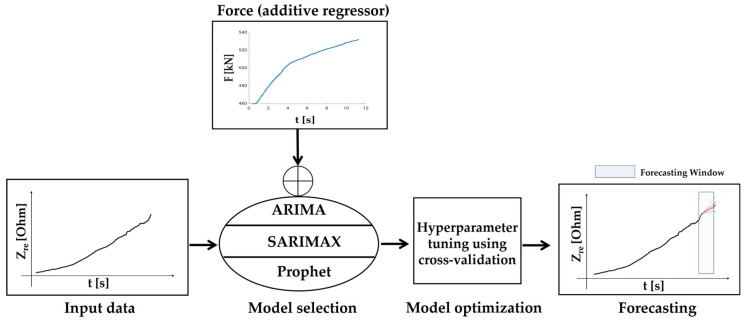
Methodology to obtain predictions based on ancillary data.

The monitoring system and the AI algorithms employed in this study leverage a cloud-based pipeline to collect and store time series data retrieved from different concrete specimens. The study objectives entail a thorough investigation to ascertain the most suitable approach for damage prediction, comparing forecasting capabilities between statistical models and AI techniques. Statistical models operate based on predefined patterns, offering reliability in forecasting; in contrast, ML approaches are proficient in handling intricate patterns within large-scale datasets. Therefore, guided by the literature, two prominent statistical methodologies, i.e., ARIMA and SARIMAX, were utilized alongside the ML approach using Prophet as part of a comprehensive analysis. ARIMA and SARIMAX operate based on predefined data assumptions, providing interpretability, while Prophet excels in handling intricate patterns in large-scale datasets. However, the latter demands greater computational resources and may lack interpretability. The comparison addresses key aspects such as scalability, interpretability, robustness, and adaptability, offering valuable insights for future research endeavours. The real part of electrical impedance was acquired with a sampling time of 0.1 s, serving as input for developing and retraining statistical and ML models (Figure 4). The corresponding values of force were additionally imported. The models and the underlying working regression mechanism is now described in detail.

The ARIMA model, a widely employed time series forecasting method, combines autoregressive (AR) and moving average (MA) components with differencing to address non-stationary time series data and is effective in capturing linear trends and seasonality in time series data. The model hyperparameters (p, d, and q) play a crucial role in forecasting future values based on historical data. The model represented as ARIMA(p, d, q) utilizes three terms (p, q, and d) to forecast future values based on historical data, as expressed in Equations (1) and (2), where p, q, and d values represent the hyperparameters for the ARIMA model imported from the *statsmodels* library in Python (v. 3.10.3).

(1) $$Y_{t}=c+\epsilon_{t}+\sum_{i=1}^{p}\;\varphi_{i}X_{t-i}+\sum_{i=1}^{q}\;\theta_{i}\epsilon_{t-i}+\delta t$$

(2) ARIMA(p,d,q)=AR(p)+I(d)+MA(q)

where

- p is the autoregressive term; it represents the relationship between an observation and past observations at multiple lag values, with higher values indicating a robust autocorrelation at various lags;
- d is the differencing term; it signifies the relationship between the current observation and past value at multiple lag values;
- q is the moving average term; it represents the connection between an observation and a residual error from a moving average model applied to lagged observations;
- *θ* and *ϕ* are coefficients associated with the AR and MA components, respectively;
- *ϵ_t_* is the error term.

A higher value of hyperparameters implies a model relying on more past observations for predicting the current value. Since ARIMA cannot incorporate seasonal effects, a newer model expands this with the additional seasonal component to it.

Seasonal ARIMA (SARIMA) with external variables, i.e., SARIMAX, is proposed for subsequent statistical methods. The model expands upon ARIMA by incorporating additional seasonal elements and external variables essential for handling periodic patterns in time series data, represented by Equation (3):

(3) $$\phi_{p}\left(L\right)\;\phi_{P}\left(Ls\right)\Delta^{d}\Delta_{s}^{d}u_{t}=A\left(t\right)+\theta \;q\left(L\right)\;\theta Q\left(Ls\right)\zeta_{t}$$

where it develops on the ARIMA by the following terms:

- *ϕ_p_*(*L*) is the autoregressive component of order p;
- *ϕ_p_*(*Ls*) is the seasonal autoregressive component of order p;
- Δ*^d^* is the non-seasonal differencing of order d;
- $\Delta_{s}^{d}$ is the seasonal differencing of order d;
- *A*(*t*) represents a deterministic trend, i.e., seasonality;
- *θq*(*L*) is the seasonal moving average component of order q;
- ζ_t_ is the seasonal error term;
- *s* is the seasonal period;
- *P* is the seasonal autoregressive component of order P;
- *D* is the seasonal differencing of order D;
- *Q* is the seasonal moving average component of order Q.

SARIMAX integrates seasonal autoregressive (P), seasonal differencing (D), and seasonal moving average (Q) terms in conjunction with non-seasonal ARIMA components (p, d, and q). This adds an ability to handle both seasonality and external factors. The seasonal autoregressive term (P) captures the connection between an observation and its seasonal lag values, considering the seasonal patterns within the dataset. Likewise, the seasonal moving average term (Q) establishes the association between an observation and the residual error derived from a seasonal moving average model applied to seasonal lagged observations. Seasonal differencing (D) is applied to the seasonal observations to ensure the seasonal stationarity of the data. By incorporating these seasonal components and non-seasonal ARIMA elements, SARIMAX comprehensively addresses both non-seasonal and seasonal patterns in time series data.

On the other hand, Prophet builds on the statistical approaches as an advanced forecasting algorithm in the realm of AI. The model was imported from the *prophet* library written in Python and it represents the time series as the sum of three components: (i) trend, (ii) seasonality, and (iii) holidays, as shown in Equation (4):

(4) y(t)=g(t)+s(t)+h(t)+ε(t)

where

- *g*(*t*) is the trend function, modelling non-periodic changes; it can be logarithmic;
- *s*(*t*) is the seasonality function, relying on the Fourier series; it provides a flexible model of periodic effects to model changes that are repeated at regular time intervals (e.g., weekly and yearly seasonality), and it is also possible to have more than one seasonality in the same series;
- *h*(*t*) represents holidays; it models irregular events that temporarily alter the time series;
- *ε*(*t*) is the error term, representing changes in the time series that the model does not capture; it is regarded as a normal distribution.

Prophet decomposes the entered time series into additive components, modelling the trends as a piecewise linear logistic growth curve. Seasonality is captured through Fourier series expansion. It can model abrupt patterns, which can be entered on a custom basis through holiday effects that are not activated in this case study.

The described models, guided by the force regressor, produce forecasts. The predicted electrical impedance values, when combined with a strategically defined acceptable threshold value, enable the implementation of an alert system (i.e., an early warning system). This system signals deviations within a specified range, enhancing the capacity for timely response and proactive damage mitigation.

### 2.5. Model Training and Hyperparameter Tuning Process

The training of the models employed a dual approach involving both model selection and tuning, guided by cross-validation. The process is illustrated in Figure 4, where the model input consists of the real value of electrical impedance (Z_Re_) and force (F) as an additive regressor. To maintain consistency in scale across features and the target variable, a MinMax scaler normalized the input data, promoting convergence during the model learning process. Following normalization, the data underwent training on the designated dataset, and cross-validation was instrumental in fine-tuning hyperparameters, which were subsequently chosen with a focus on minimizing errors. While ARIMA and SARIMAX models necessitate the manual implementation of cross-validation techniques, for Prophet it is possible to leverage the built-in functions, streamlining the process and enhancing efficiency. These refined hyperparameters played a crucial role in generating accurate and optimized forecasts. The dataset was split into training (90%) and testing (10%) sets. 

The training dataset serves as an instructional set, enabling the model to learn intricate patterns and relationships. In addition, a cross-validation technique was implemented, where 10% of the training data were designated as a validation set to assess the model performance, aiding in the tuning of hyperparameters and preventing overfitting by evaluating model generalization. The model was trained on a predetermined initial training set, progressively expanding its size at regular intervals during each cross-validation fold. The size of the folds was determined by two key parameters, namely period and horizon. The period parameter dictates the length of a seasonal cycle, while the horizon parameter defines the duration for future predictions. Predictions were made for the defined horizon length during each fold, and this iterative process was applied across the entire time series spanning the training and validation set, ensuring a thorough evaluation of the model performance across diverse data segments. The model undergoes training on an initial set in each cross-validation fold, gradually expanding the training size every period. We use 50% of the length of the validation set as the horizon and 50% of the horizon as the period, as per suggestions of the model documentation. Predictions are then made for the defined horizon length, iterating as set by the period, across the validation set to obtain the most optimal hyperparameters. This iterative process provides a robust evaluation of the model performance across various data segments. These hyperparameters are then used to evaluate the model performance and its ability to generalize on testing datasets. Post-forecasting, the denormalization process was applied to restore the results to their original scale. This step was essential for the meaningful interpretation and practical application of the results. The forecast in terms was performed over 10% of the time series, and the performance was evaluated through the following metrics:

- Mean Absolute Error (MAE), which is the absolute value of the difference between the paired accurate and predicted data;
- Mean Absolute Percentage Error (MAPE), which is the percentage expression of MAE obtained through the normalization of the real data;
- Root Mean Square Error (RMSE), which is the average difference between the predicted and real data;
- Correlation, which is the strength and direction of the linear relationship between the predicted and the actual values.

## 3. Results and Discussion

In this section the results related to electrical impedance measurements and crack aperture assessment are reported, as well as those related to prediction models. 

### 3.1. Electrical Impedance Measurements

**Figure 5:**
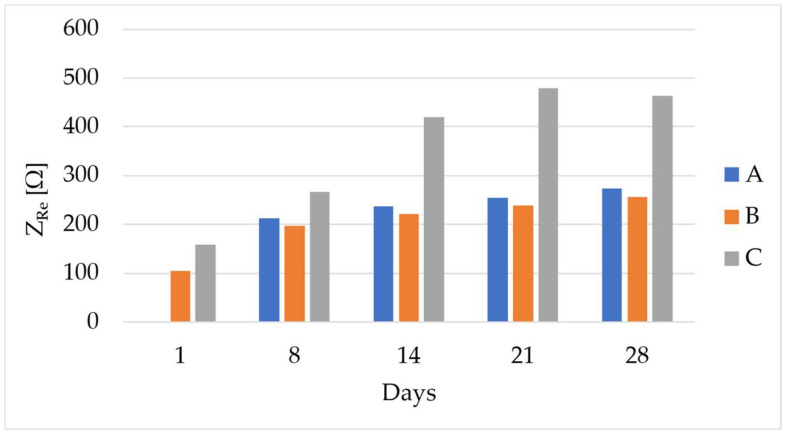
Electrical impedance values (real part) monitored during curing on the tested specimens (i.e., A, B, and C).

**Figure 6:**
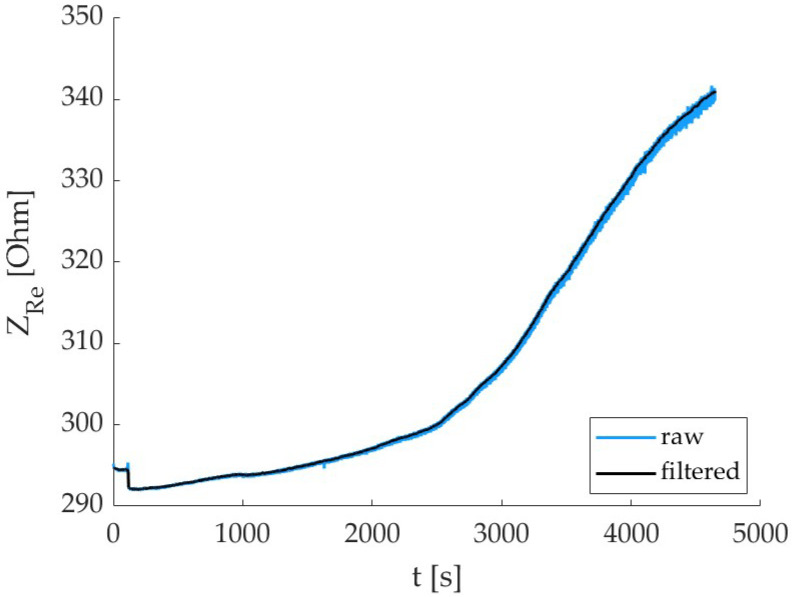
Filtering process of the real part of the electrical impedance signal (raw signal in blue color, filtered one in black).

The electrical impedance values monitored during curing are reported in Figure 5. As expected, the trend is increasing during to the material hydration process; the inter-specimen variability is high, and this is due to the inner nature of concrete. An example of the effect of the filtering procedure on the electrical impedance signal is reported in Figure 6; the noise is significantly removed, and this allows for a better evaluation of the correlation with the force signal as well as enhanced data quality for the prediction models.

**Figure 7:**
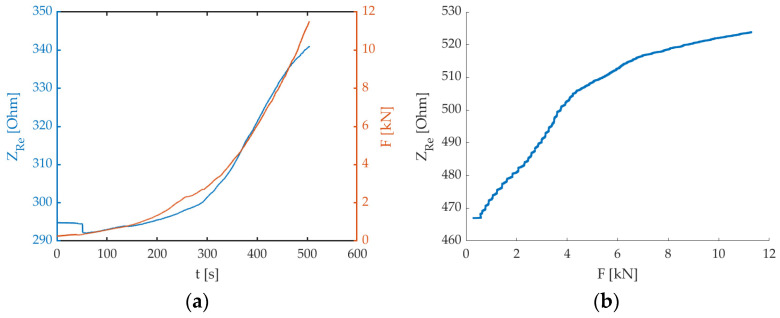
Examples of (**a**) real part of electrical impedance (Z_Re_—blue curve) and force (F—orange curve) signals over time (Specimen B, t1 test time), and (**b**) real part of electrical impedance (Z_Re_) vs. force (F) signals (Specimen C, t2 test time).

When the flexural load is applied, in a perpendicular direction with respect to the electrodes array, the electrical impedance increases since the inter-electrode spacing widens. This can be observed in the graph of the real part of electrical impedance (Z_Re_) together with the force (F) signals over the test interval (Figure 7a). At the beginning of the trial there is a decrease in the electrical impedance, possibly because the initial load application compresses the sensing volume, hence easing the passage of the electrical current (maybe voids are collapsed). Then, the flexure of the beam element prevails and affects the electrode array, therefore both Z_Re_ and F signals show a similar increasing trend. The overall correlation between the two signals is very good, as can be verified in Table 2. There is no specific trend over time and, given the very high values obtained, we can consider the results comparable among test times, indicating that the real part of electrical impedance follows very efficiently with the applied load both on intact specimens and in the case of the cracking phenomena that has already happened. Hence, the piezoresistivity property of the material is very good.

**Figure 8:**
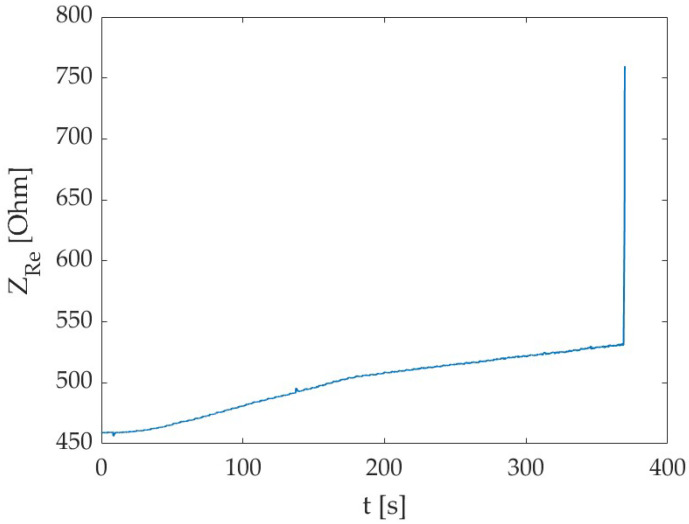
Real part of electrical impedance (Z_Re_) over time, including the crack formation (i.e., final high peak) (Specimen C, t2 test time).

Observing the graphs of the correlation between the electrical impedance and the applied force (Figure 7b), it is possible to evidence a sensitivity decreasing at higher force values. Hence, we can infer that the degradation/cracking phenomena occurring in the specimen somehow impacts on the method sensitivity towards the external load, probably because voids are forming and this hinders the passage of the electrical current. This means that the system promptly detects the applied load, but it could be not so reactive in sensing changes if the load is maintained for a prolonged time and increases slowly (but this is not the case for earthquake-related loads). However, it is possible to confirm the suitability of the method to monitor external loading and promptly identify crack formation. Indeed, when a crack has formed, we can notice a very high peak in the electrical impedance, due to the complete interruption of the electrical path (Figure 8).

### 3.2. Assessment of Crack Aperture

**Figure 9:**
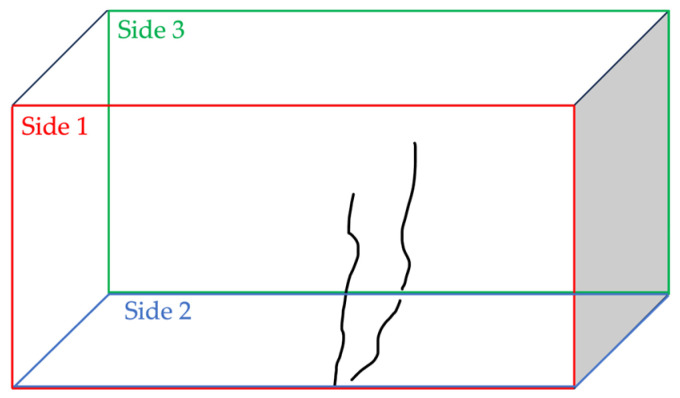
Specimen pattern: the defect is distributed on side 1 (front-red), side 2 (bottom), and side 3 (back-green).

The specimen was analyzed in terms of crack aperture considering the defects appearing on the specimen sides as identified in Figure 9. 

**Figure 10:**
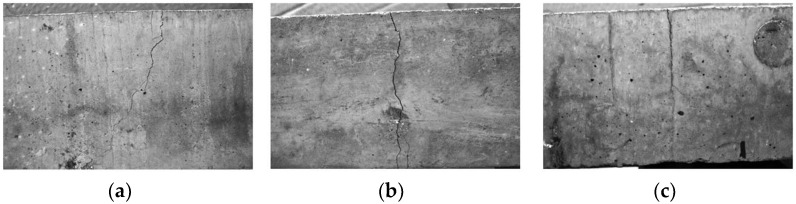
Example of images of the cracked specimen on (**a**) side 1, (**b**) side 2, and (**c**) side 3.

An example is shown in Figure 10, where the defect evaluation is performed for each side where the discrepancy appears.

**Figure 11:**
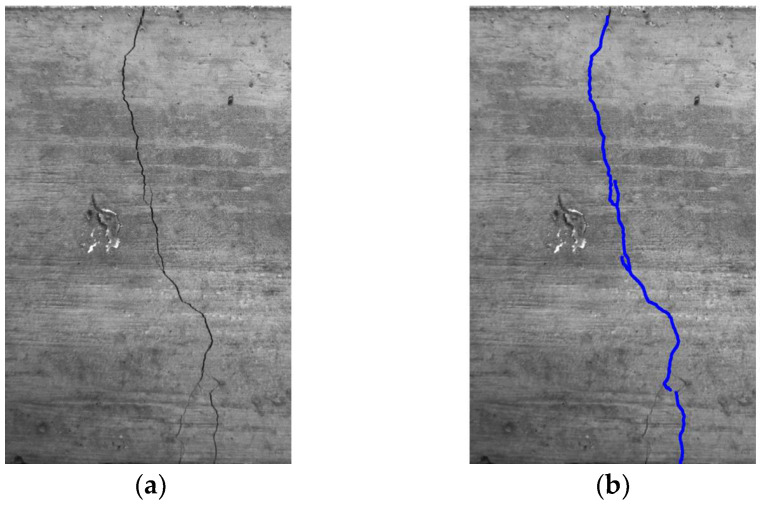
Example of (**a**) cracked specimen and related (**b**) crack identification (blue line represents the detected crack).

Considering a single image, the quantified value represents the mean width of the crack, determined along the identified curvilinear abscissa. An exemplification of a demarcated fissure is depicted in Figure 11. 

**Figure 12:**
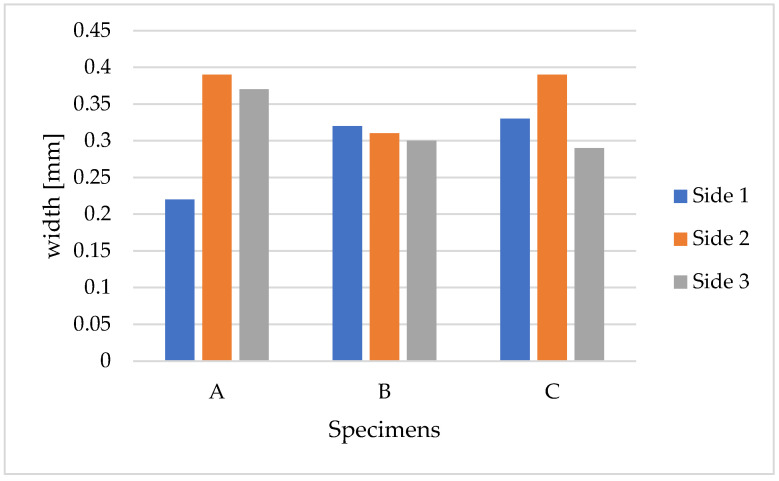
Crack width measurement for each side of each specimen (A, B, and C).

The result reports three distinct measurements per specimen, corresponding to the calculated widths for each observed face, detailed in Figure 12.

Defect assessment, illustrated in the previous lines, was performed using a computer vision tool aligned with the state-of-the-art [23]. In fact, defect identification and segmentation was executed using a neural network (i.e., UNet) but with a different defect measurement method, based on Steger’s algorithm [30], with respect to the method of Wu et al. [23]. Moreover, as performed in [23], the depth coordinates were also used. As stated above, the accuracy of the employed method is sub-pixel.

### 3.3. Predictive Crack Detection AI Models Based on Electrical Impedance Data

Table 3 displays the performance metrics of the tested forecasting models, i.e., ARIMA, SARIMAX, and Prophet, applied to predict the real part of the electrical impedance of specimens A, B, and C at test times t2 and t3 as per the discussed methods. In fact, at time t1, the forecasting algorithms were not employed since no cracks or fractures had formed in the specimens due to an applied force lower than the fracture load. For specimen A at t2, Prophet outperforms ARIMA and SARIMAX with lower MAE, RMSE, and MAPE, as well as a higher correlation. This trend continues for t3. Similarly, for specimens B and C, Prophet consistently exhibits superior performance compared to ARIMA and SARIMAX across the evaluated metrics and test times. Notably, at t3, ARIMA and SARIMAX show a substantial decline in performance for specimen A, with negative correlations indicating poor predictive accuracy. In contrast, Prophet maintains reliable predictions across all scenarios.

Looking at each scenario independently, at test time t2 for specimen A the ARIMA model produces an MAE of 1.19 Ω and an RMSE of 1.41 Ω. The MAPE is low (0.34%), reflecting a small percentage of prediction error. The correlation is high (98.50%), indicating a strong linear relationship between predicted and observed values. In comparison, the SARIMAX model for specimen A at t2 outperforms ARIMA with lower MAE (0.75 Ω), RMSE (0.84 Ω), and MAPE (0.21%), along with a high correlation (97.79%), slightly lesser than ARIMA. The Prophet model excels further, achieving the lowest MAE (0.51 Ω), RMSE (0.54 Ω), and MAPE (0.15%) among the models, along with a high correlation (98.91%). This suggests that Prophet provides more accurate and precise predictions for specimen A at test time t2 compared to ARIMA and SARIMAX. Moving to test time t3, the ARIMA model for specimen A exhibits higher MAE (1.06 Ω), RMSE (1.13 Ω), and MAPE (0.26%) compared to t2, and the correlation drops to 96.48%, indicating a decrease in predictive performance. The SARIMAX model also shows a decline in performance at t3 with higher MAE (1.47 Ω), RMSE (1.91 Ω), and MAPE (0.36%), along with a negative correlation (−96.48%), suggesting a reversal in the predicted and observed trends. In contrast, the Prophet model maintains its accuracy at t3 for specimen A, demonstrating the lowest MAE (0.34 Ω), RMSE (0.40 Ω), and MAPE (0.09%), along with a high correlation (96.84%).

For specimen B, at test time t2 the ARIMA model yields predictions with an MAE of 1.47 Ω, an RMSE of 1.61 Ω, an MAPE of 0.42%, and a high correlation of 99.35%. In comparison, the SARIMAX model demonstrates improved performance with lower MAE (0.25 Ω), RMSE (0.32 Ω), and MAPE (0.07%), coupled with a high correlation (99.35%). The Prophet model excels further, achieving the lowest MAE (0.21 Ω), RMSE (0.28 Ω), and MAPE (0.06%) among the models while maintaining a high correlation (99.56%). This indicates that both SARIMAX and Prophet outperform ARIMA for specimen B at test time t2, with Prophet showing the most accurate and precise predictions with a slight compromise on correlation. Moving to test time t3, the ARIMA model for specimen B exhibits higher MAE (0.66 Ω), RMSE (0.76 Ω), and MAPE (0.17%) compared to t2, and the correlation drops to 83.89%. The SARIMAX model also shows a decline in performance at t3, with higher MAE (0.92 Ω), RMSE (1.21 Ω), and MAPE (0.24%), along with a correlation of 84.03%. In contrast, the Prophet model maintains accuracy at t3 for specimen B, demonstrating the lowest MAE (0.23 Ω), RMSE (0.508 Ω), and MAPE (0.06%), along with a high correlation (83.869%).

For specimen C, at test time t2 the ARIMA model achieves an MAE of 1.21 Ω, RMSE of 1.33 Ω, a low MAPE of 0.23%, and a high correlation (98.93%). The SARIMAX model performs similarly well, with a lower MAE (1.46 Ω), RMSE (1.61 Ω), and MAPE (0.27%), along with a high correlation (98.90%). The Prophet model stands out, achieving the lowest MAE (0.64 Ω), RMSE (0.70 Ω), and MAPE (0.12%) among the models, coupled with a high correlation (98.69%). At test time t3, the ARIMA model for specimen C shows a higher MAE (35.34 Ω), RMSE (99.04 Ω), and MAPE (2.38%) compared to t2, and the correlation drops to −5.31%, indicating a significant decline in predictive accuracy. The SARIMAX model also exhibits a decline in performance at t3 with a higher MAE (43.56 Ω), RMSE (103.80 Ω), and MAPE (3.0%), along with a correlation of −9.32%. In contrast, the Prophet model maintains accuracy at t3 for specimen C, demonstrating the lowest MAE (11.65 Ω), RMSE (12.33 Ω), and MAPE (0.93%), along with a positive correlation of 5.038%.

**Figure 13:**
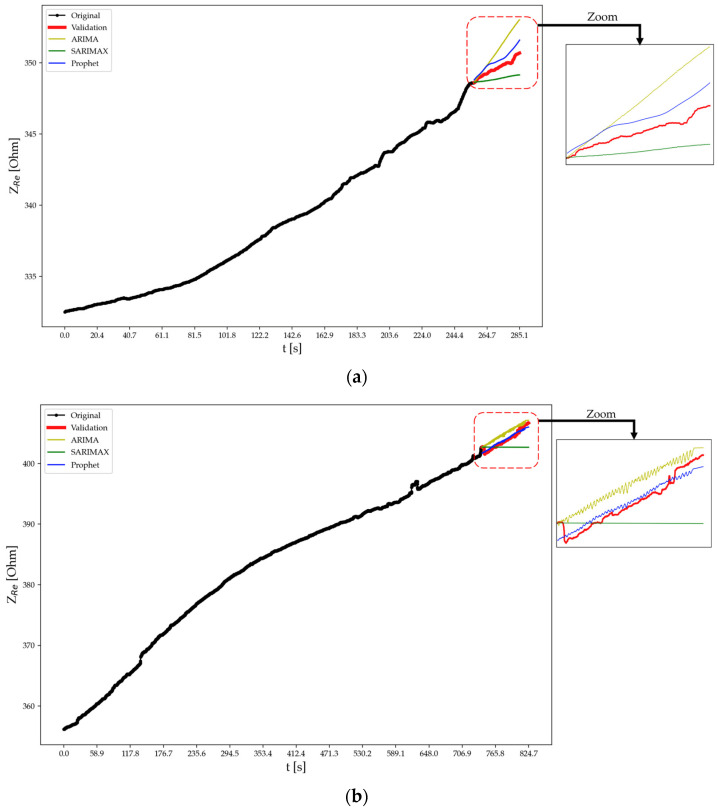
Forecasting using different approaches for specimen A at test time (**a**) t2 and (**b**) t3.

**Figure 14:**
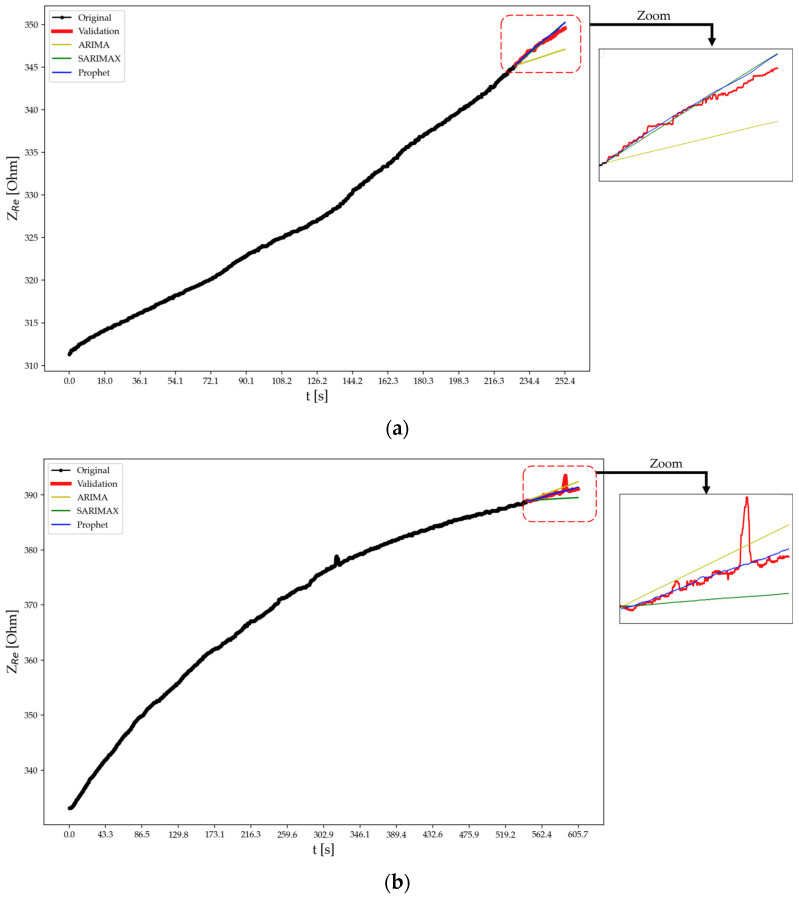
Forecasting using different approaches for specimen B at test time (**a**) t2 and (**b**) t3.

**Figure 15:**
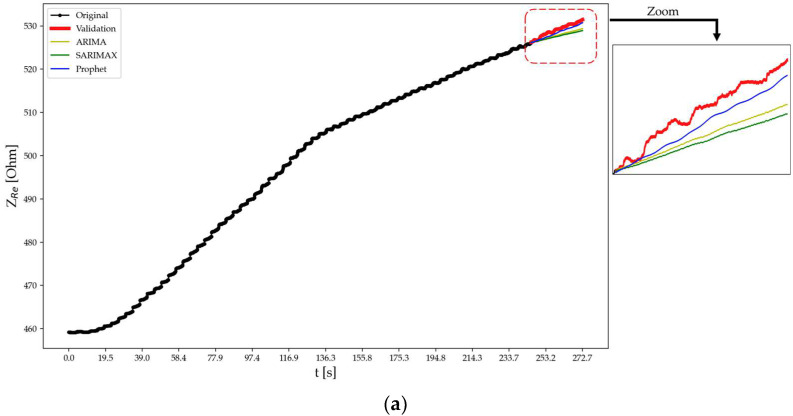

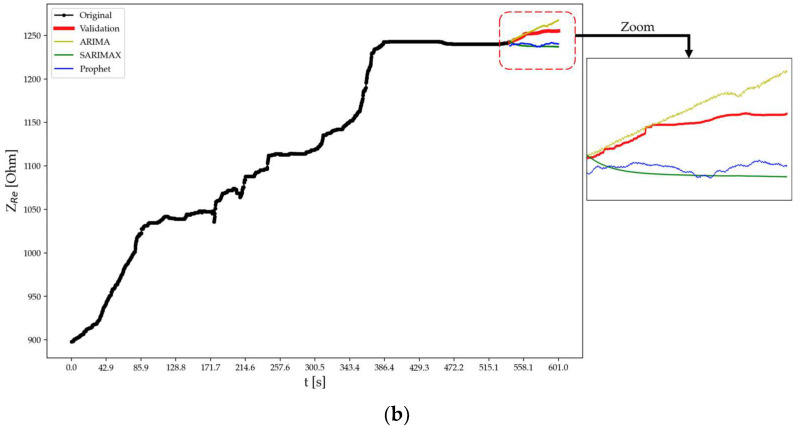
Forecasting using different approaches for specimen C at test time (**a**) t2 and (**b**) t3.

The visual examination suggests that Prophet’s predictions align more closely with the intricate dynamics of the original data, showcasing its versatility and effectiveness in handling complex temporal patterns. Upon visually inspecting the forecasts as shown in Figure 13, Figure 14 and Figure 15, it becomes evident that Prophet has effectively encapsulated the essential characteristics of the original data. The model employs a sophisticated modeling framework that incorporates the Fourier series for handling seasonality and additive components to accommodate holidays and trends. The rationale behind each prediction, therefore, is dependent on the interplay and inherent non-linearities within these components, introducing a level of complexity that diverges from traditional statistical approaches. In contrast, both SARIMAX and ARIMA, owing to their strongly linear nature in handling certain test cases, tend to generate predictions in the form of straight lines, ultimately failing to capture any underlying seasonality or intricate patterns present in the data. However, Prophet not only demonstrates promising results in quantitative metrics, but also stands out for its ability to capture nuanced patterns. The obtained results are aligned with the current state-of-the-art literature [26,27,29], confirming that AI models like Prophet exhibit superiority over traditional machine learning models such as ARIMA and SARIMAX, particularly in the intricate task of forecasting within complex datasets where external variables are present. This reinforces the growing consensus on the efficacy of advanced AI approaches in handling the intricacies of contemporary datasets for more accurate and nuanced predictions.

## 4. Conclusions

The present work focused on the exploitation of self-sensing materials and related sensors for the measurement of electrical impedance for a potential distributed sensor network for buildings in seismic areas. Such an approach supports the development of early warning systems relying on the continuous monitoring of the structure, which are capable of promptly highlighting adverse events, potentially mining the safety and durability of a certain element thanks to the employment of AI techniques.

In particular, the results show the following:

- Electrical impedance measurements can follow the trend of applied external loads (correlation > 0.9), timely evidencing the occurrence of cracking phenomena;
- Self-sensing materials (obtained with the addition of biochar and recycled carbon fibers) promote the piezoresistive ability of cement-based materials, hence making a structure able to perceive its own structural health condition;
- Vision-based techniques can be seen as an effective inspection method, accurately quantifying damage that can be identified through monitoring techniques (e.g., electrical impedance measurements);
- AI models can significantly enhance the ability to forecast and monitor complex systems, as evidenced by the comparison in predicting the real part of electrical impedance. AI-based models demonstrate superior performance in quantitative metrics and can capture intricate patterns. The forecasts, when combined with strategically defined threshold values, potentially enable the implementation of an effective early warning system, which is pivotal in signaling deviations in electrical impedance within acceptable ranges (depending on the material mix design), empowering timely responses and proactive measures to mitigate potential damages.

Thus, the proposed solution can be integrated in a monitoring platform, effectively supporting remote decision-making processes; in particular, in seismic areas where it is fundamental to monitor external forces, potentially mining the structural health of a certain building. This can assist decision makers in the management of emergency situations, hence improving the resilience not only of the building but of the whole community facing the natural hazards. The proposed methodology could also be extended and scaled to different types of natural hazards (e.g., floods or heat island), provided that suitable sensors (accurate and low-cost at the same time) are installed in the structure to be monitored and that proper data processing approaches are adopted to highlight the features of interest for the specific application. Indeed, the challenge is to try to identify the features that are better correlated with an external variable of interest, such as the external force in seismic areas, presence of water during floods, or temperature in heat islands. It is worthy to note that a stable Internet connection as well as power supply should be guaranteed for remote access to data, especially in real-time. Moreover, with the system being wired, care is needed for system preservation, and sporadic maintenance may be needed.

## Figures and Tables

**Table 1:** Mix design of concrete specimens.

Cement [kg/m^3^]	Water [kg/m^3^]	Air [%]	Sand [kg/m^3^]	Intermediate Gravel [kg/m^3^]	Coarse Gravel [kg/m^3^]	RCF [kg/m^3^]	BCH [kg/m^3^]
470.0	235.0	2.5	795.0	321.0	476.0	0.9	10.0

**Table 2:** Correlation coefficient between electrical impedance and force signals.

Specimen	Test Time	Correlation Coefficient
A	t1	0.89
	t2	0.98
	t3	0.99
B	t1	0.99
	t2	0.97
	t3	0.98
C	t1	0.99
	t2	0.96
	t3	0.97

**Table 3:** Standard quantitative evaluation metrics for forecasting model comparison.

		Specimen											
		A				B				C			
Test Time	Approach	MAE (Ω)	RMSE (Ω)	MAPE (%)	Correlation (%)	MAE (Ω)	RMSE (Ω)	MAPE (%)	Correlation (%)	MAE (Ω)	RMSE (Ω)	MAPE (%)	Correlation (%)
t2	ARIMA	1.19	1.41	0.34	98.50	1.47	1.61	0.42	99.35	1.21	1.33	0.23	98.93
	SARIMAX	0.75	0.84	0.21	97.79	0.25	0.32	0.07	99.35	1.46	1.61	0.27	98.90
	Prophet	0.51	0.54	0.15	98.91	0.21	0.28	0.06	99.56	0.64	0.69	0.12	98.69
t3	ARIMA	1.06	1.13	0.26	96.48	0.66	0.76	0.17	83.89	35.34	99.04	2.38	−5.31
	SARIMAX	1.47	1.91	0.36	−96.48	0.92	1.20	0.24	84.03	43.56	103.80	3.00	−9.32
	Prophet	0.34	0.40	0.09	96.84	0.23	0.51	0.06	83.87	11.65	12.33	0.93	5.04

## Data Availability

The data presented in this study are available on request from the corresponding author.
